# Supplementary material for: N-Doped Porous Carbon-Nanofiber-Supported Fe3C/Fe2O3 Nanoparticles as Anode for High-Performance Supercapacitors
Source: Molecules. 2023 Jul 30;28(15):5751. doi: 10.3390/molecules28155751 (PMC10421154; doi:10.3390/molecules28155751)
Supplement: Supplementary file 1 [file molecules-28-05751-s001.zip › molecules-2463859-supplementary.pdf]

N-Doped Porous Carbon-Nanofiber-Supported Fe_3C/Fe_2O_3 Nanoparticles as Anode for High-Performance Supercapacitors

Li Li [1], Fengting Xie [1], Heyu Wu [1], Yuanyuan Zhu [1,2], Pinghua Zhang [1,*], Yanjiang Li [1,3,4], Hengzheng Li [1], Litao Zhao [1] and Guang Zhu [1,*]

1 Key Laboratory of Spin Electron and Nanomaterials of Anhui Higher Education Institutes, Suzhou University, Suzhou 234000, China; lili@ahszu.edu.cn (L.L.); coco9024137@163.com (F.X.); wuheyu19991105@163.com (H.W.); zhuyy@dicp.ac.cn (Y.Z.); yjli@ahszu.edu.cn (Y.L.); lihengzheng@ahszu.edu.cn (H.L.); ltzhao@ahszu.edu.cn (L.Z.)
2 State Key Laboratory of Catalysis, Dalian Institute of Chemical Physics Chinese Academy of Sciences, Dalian 116023, China
3 School of Chemistry and Materials Science, University of Science and Technology of China, Hefei 230026, China
4 Institute for Carbon Neutralization, College of Chemistry and Materials Engineering, Wenzhou University, Wenzhou 325035, China
* Correspondence: zphchemical@163.com (P.Z.); guangzhu@ahszu.edu.cn (G.Z.)

1 Electrochemical measurements

All of the electrochemical measurements were carried out on a CHI660E electrochemical workstation. In a three-electrode configuration, a standard Hg/HgO was used as the reference electrode, a slice of Pt foil was acted as the counter electrode. The work electrode was prepared by coating the homogeneous slurry containing active material, carbon black and PVDF (5 wt.% NMP solution) in a ratio of 8:1:1 onto a piece of graphite paper and dried at 80 °C for 24 h. The single electrode measurements were conducted in 2 mol L^{-1} KOH aqueous electrolyte. The electrochemical impedance spectroscopy (EIS) was performed in the frequency range from 0.01 to 100 kHz with an amplitude of 10 mV at open circuit potential. Cyclic voltammetry (CV) and galvanostatic charge-discharge (GCD) were conducted at room temperature. The specific capacitances derived from GCD (C_g, F g^{-1}) results were calculated by means of equation (S1):

$$C_g = \frac{i\Delta t}{m\Delta V} \quad \text{(S1)},$$

where i, Δt, m and ΔV are the current density (A), discharge time (s), mass of active material (g), and

voltage window (V), respectively.

The asymmetric supercapacitor (SC) was assembled using N-doped carbon nanofibers supporting Fe_3C/Fe_2O_3 nanoparticles hybrid architecture composite (NCFCO) anode and activated carbon (AC) cathode and the electrochemical performances were measured in a two-electrode system in 2 mol L^{-1} KOH aqueous electrolyte. The masses of active material in the anode (m_-, g) and cathode (m_+, g) were determined according to the following equation:

$$\frac{m_+}{m_-} = \frac{C_- \Delta V_-}{C_+ \Delta V_+} \quad \text{(S2)},$$

where C_+ (F g^{-1}) and C_- (F g^{-1}) are the specific capacitances, ΔV_+ (V) and ΔV_- (V) are the voltage windows of anode and cathode, respectively. The energy density (E, Wh kg^{-1}) and power density (P, W kg^{-1}) of the as-assembled device were computed in terms of equation (S3) and (S4) individually:

$$E = \frac{C \Delta V^2}{7.2} \quad \text{(S3)},$$

$$P = \frac{E}{\Delta t} \quad \text{(S4)}.$$

2 Characterizations

The morphology was characterized on a scan electron microscope (SEM, Hitachi S1510) equipped with energy dispersive X-ray spectroscopy (EDS, Oxford). The phase structure was examined by X-ray diffraction (XRD, Rigaku, Smartlab) and Raman spectroscopy (Horiba, Xplora Plus). The elemental composition and chemical valence on the surface of the samples were tested by X-ray photoelectron spectroscopy (XPS, Escalab 250Xi). The specific surface area (SSA) and pore size distribution were calculated on a physical analyzer (Micromeritrics, ASAP 2020 Plus) applying Brunauer-Emmett-Teller (BET) method and Barrett-Joyner-Halenda (BJH) model based on N_2 adsorption-desorption isotherms at 77 K, respectively.

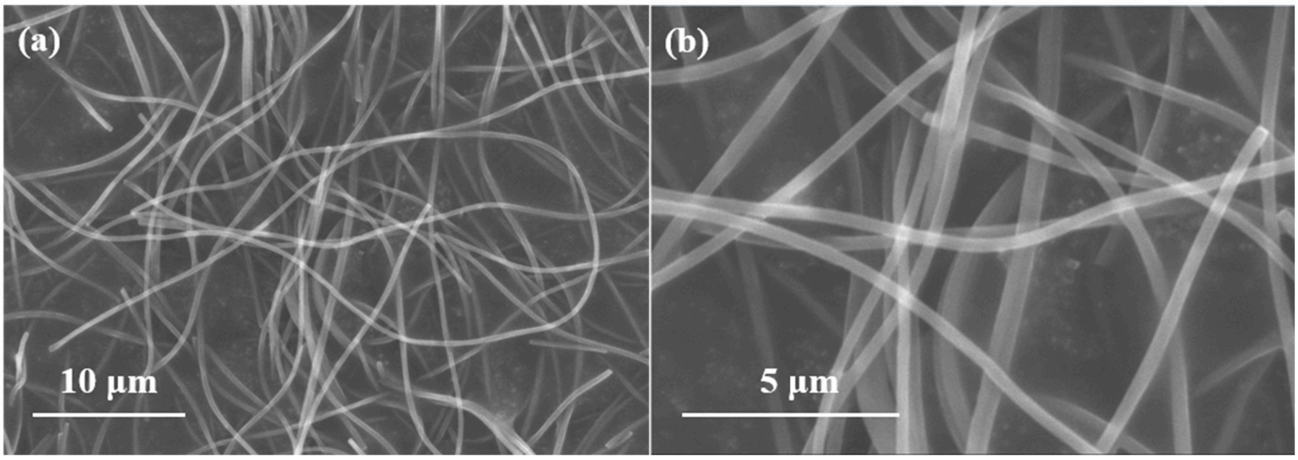

Figure S1 Low (a) and High (b) magnification SEM images of the electrospinning nanofiber precursor before annealing.

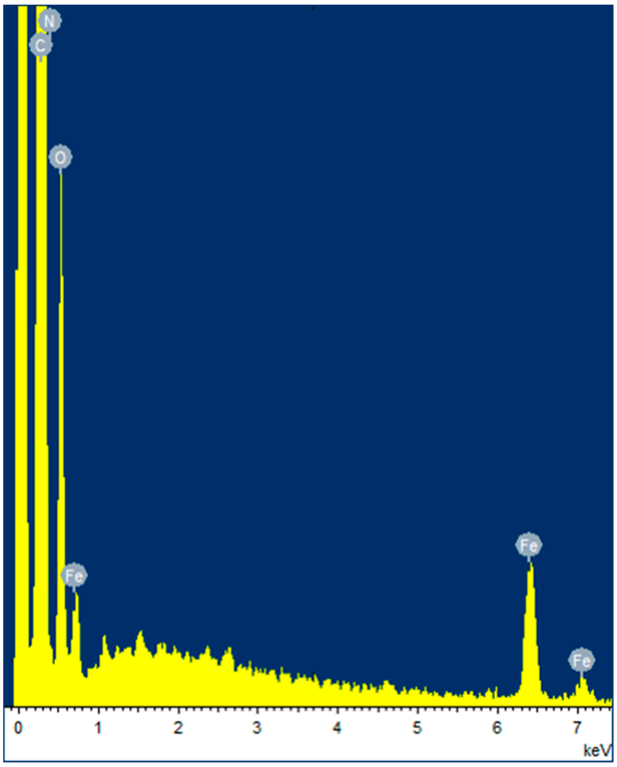

Figure S2 EDS spectrum of NCFCO.

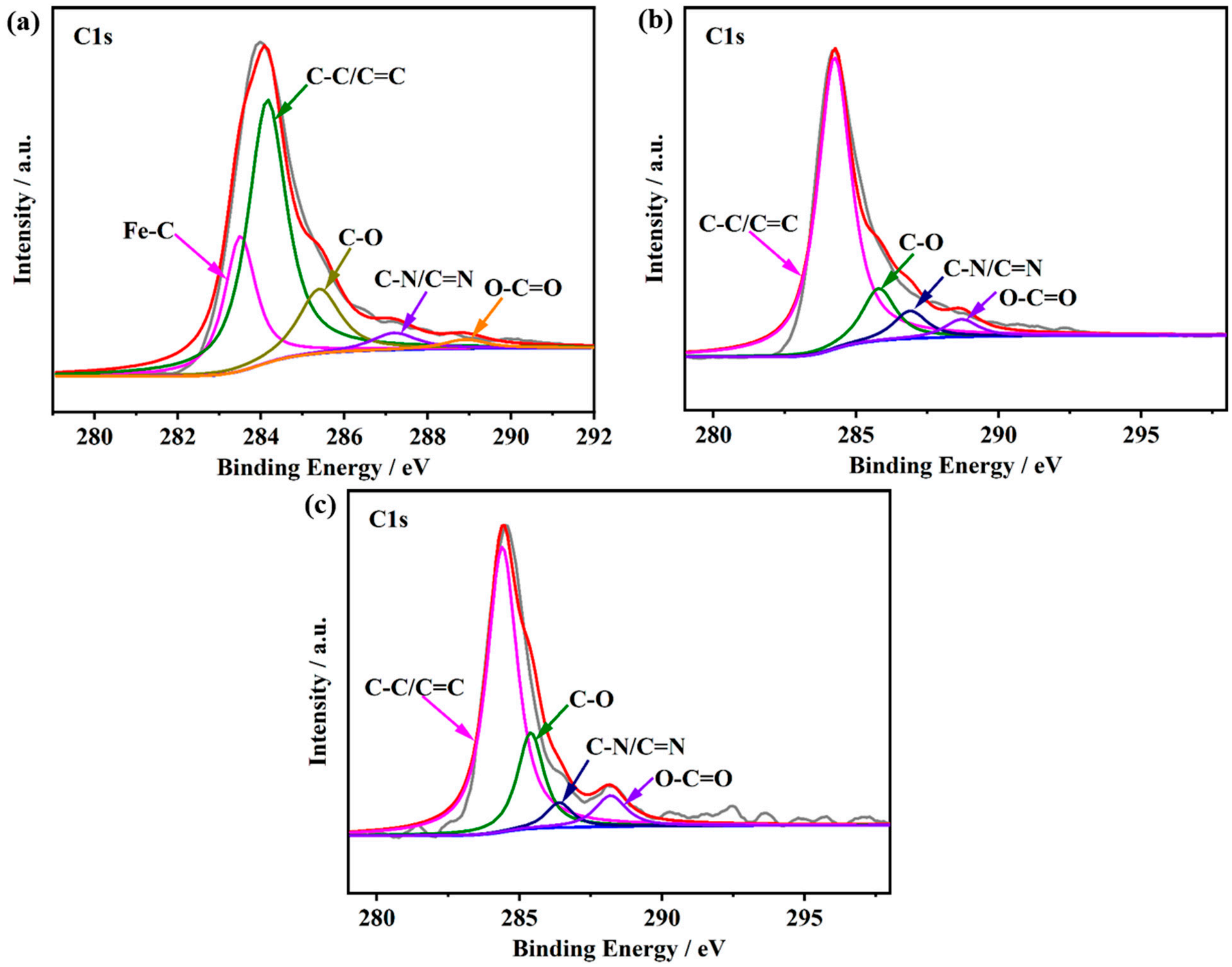

Figure S3 High resolution C1s spectra of NCFC (a), NCFCO (b) and NCFO (c).

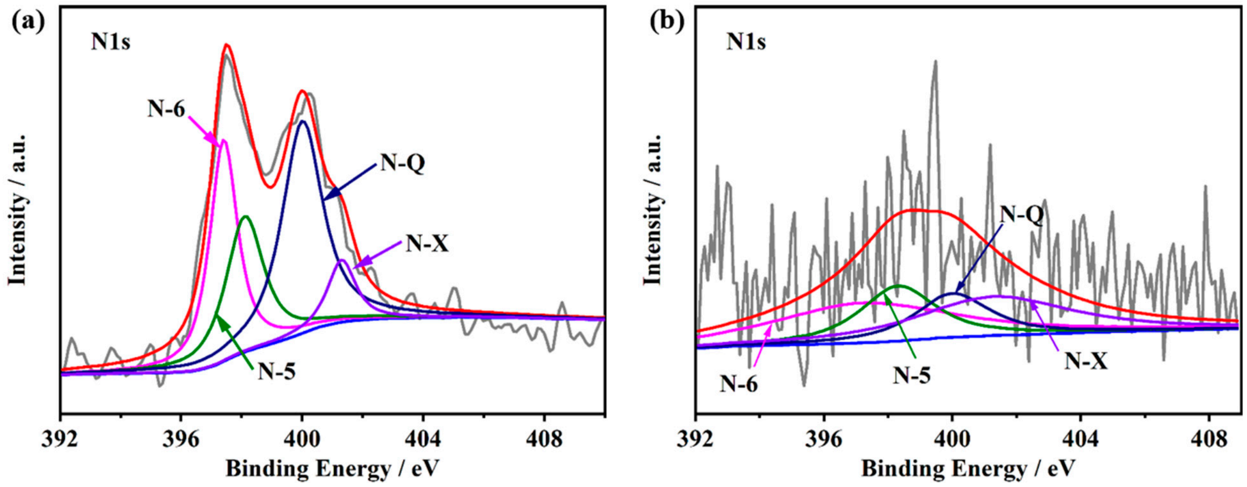

Figure S4 High resolution N1s spectra of NCFC (a) and NCFO (b).

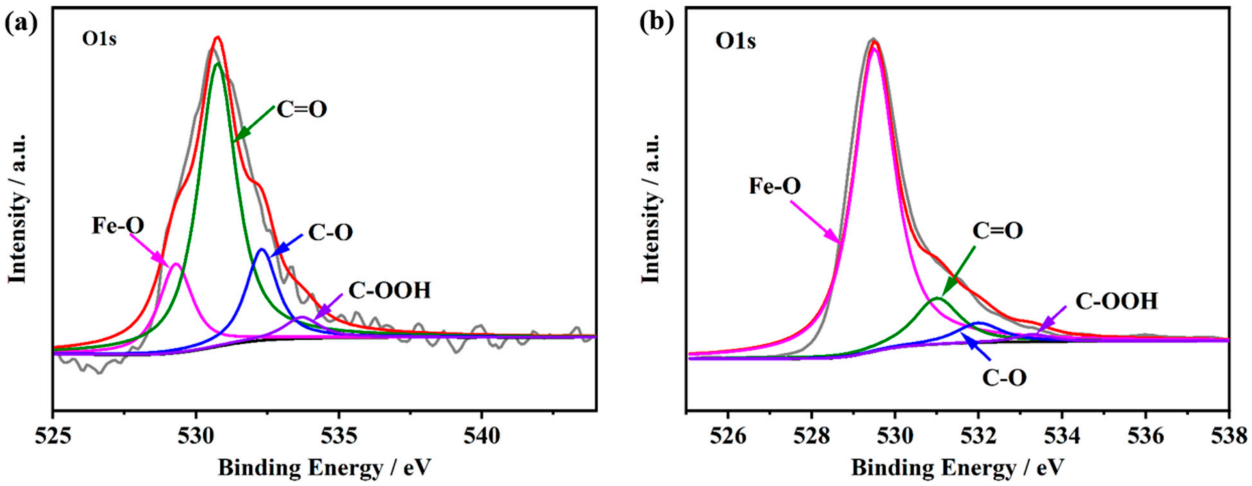

Figure S5 High resolution O1s spectra of NCFC (a) and NCFO (b).

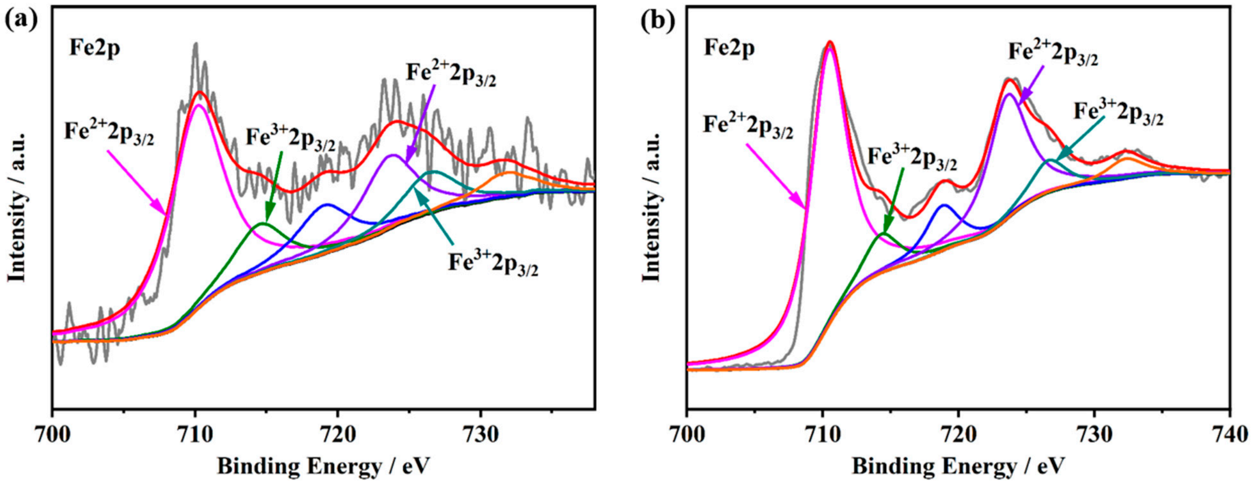

Figure S6 High resolution Fe2p spectra of NCFC (a) and NCFO (b).

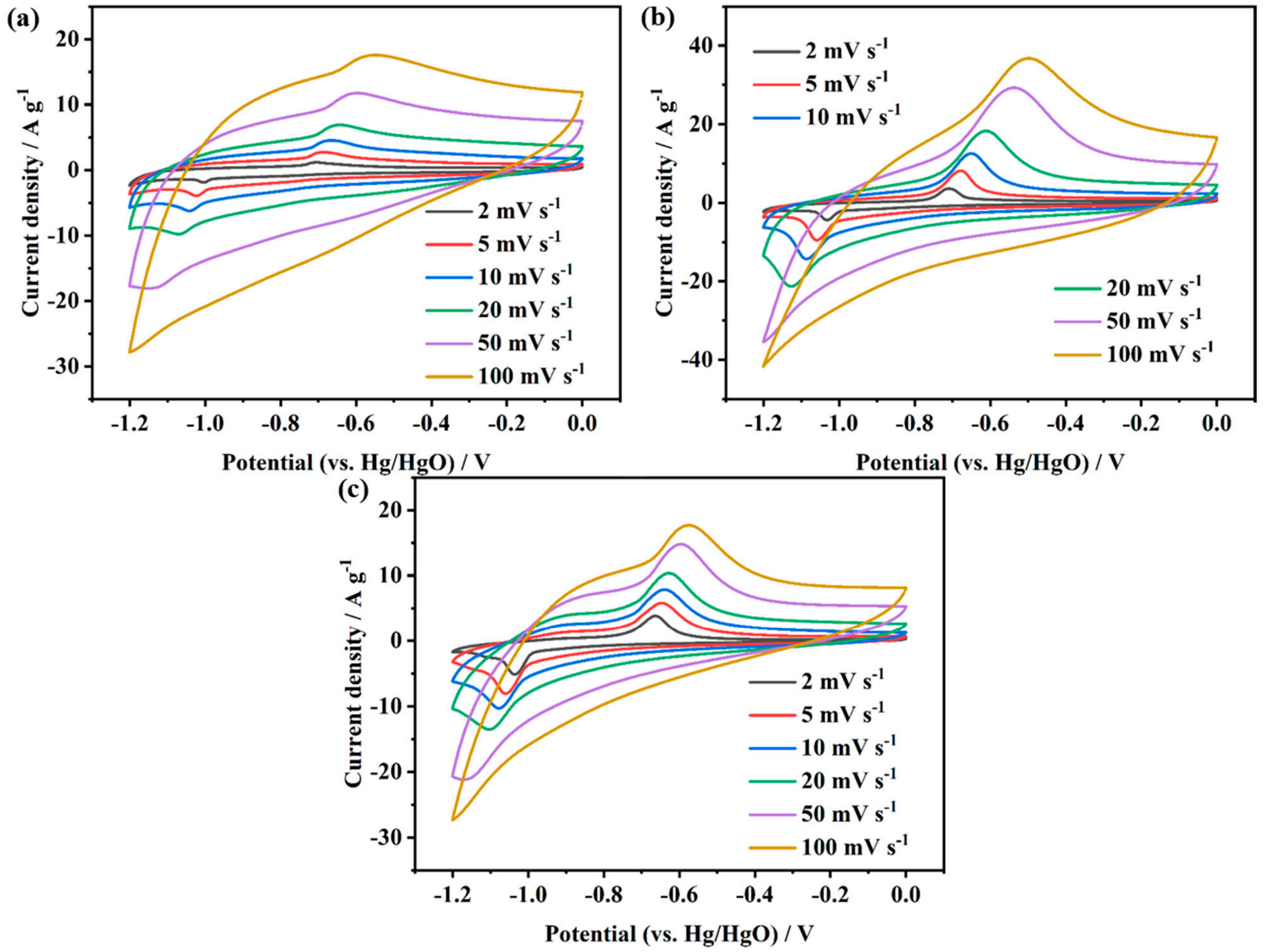

Figure S7 CV curves of NCFC (a), NCFCO (b) and NCFO (c) at different scan rates.

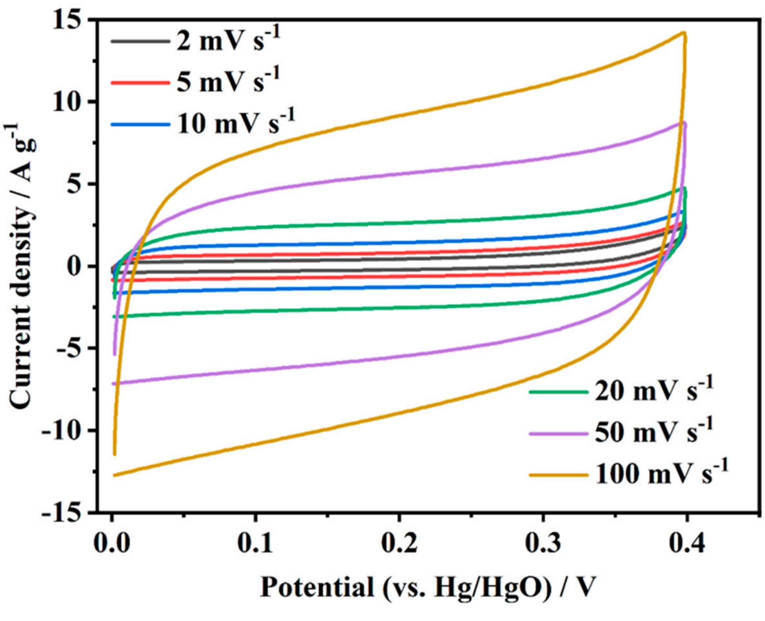

Figure S8 CV curves of AC at different scan rates.

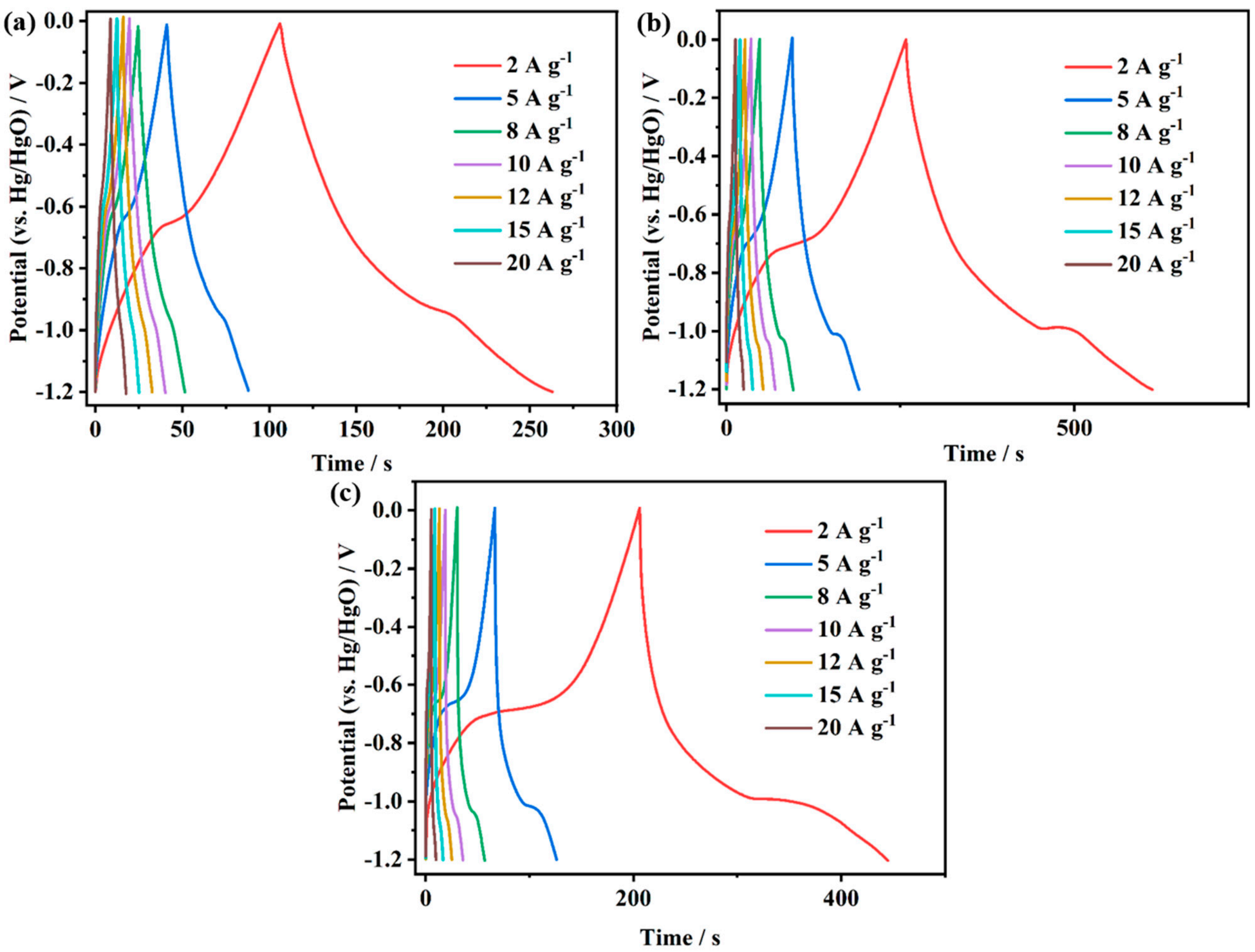

Figure S9 GCD curves of NCFC (a), NCFCO (b) and NCFO (c) at different current densities.

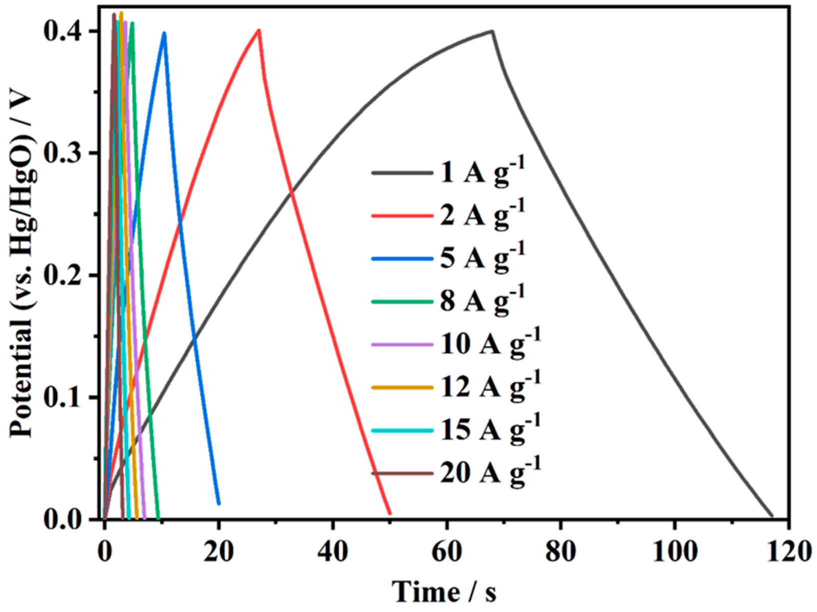

Figure S10 GCD plots of AC at different current densities.

Table S1. SSA, pore volume and average pore diameter of NCFC, NCFCO and NCFO.

	NCFC	NCFCO	NCFC
SSA / m^2 g^{-1}	215.9	210.7	21.6
Pore volume / cm^3 g^{-1}	0.136	0.124	0.078
Average pore diameter / nm	1.85	1.97	11.8

Table S2. Specific capacitances of NCFCO and previously reported Fe-based oxides/carbides.

Materials	Current density / A g^{-1}	Specific capacitance / F g^{-1}	Reference
Fe_xC_y/Fe/carbon nanofibers	1	340	[1]
Fe_2O_3/Fe_3C/N-doped carbon nanosheet	0.5	240	[2]
Fe_2O_3/MXene	1	486.3	[3]
Carbon coated Fe_3O_4/carbon cloth	1	463	[4]
Fe_3O_4/N-doped carbon nanosheets	0.5	522.7	[5]
Fe_3O_4/graphene/carbon cloth	1	406	[6]
Fe_2O_3/carbon	1	221.5	[7]
Fe_2O_3/MXene	1	182	[8]
Carbon/Fe/Fe_2O_3	0.1	177	[9]
Fe_3C/N-doped carbon	2	325	[10]
NCFCO	2	590.1	This work

References

1. Chen, L.; Liang, B.; Lv, J.; Chen, M.; Hu, J.; Zeng, K.; Yang, G. Route to a porous carbon nanofiber membrane containing Fe_xC_y/Fe by facile in situ ion-exchange functionalization of the PAA carboxyl group: exemplified by a supercapacitor. *ACS Appl. Energy Mater.* **2022**, *5*, 1580-1594.
2. Cheng, J.; Wu, D.; Wang, T. N-doped carbon nanosheet supported Fe_2O_3/Fe_3C nanoparticles as efficient electrode materials for oxygen reduction reaction and supercapacitor application. *Inorg. Chem. Commun.* **2020**, *117*, 107952.
3. Shi, T.-Z.; Feng, Y.-L.; Peng, T.; Yuan, B.-G. Sea urchin-shaped Fe_2O_3 coupled with 2D MXene nanosheets as negative electrode for high-performance asymmetric supercapacitors. *Electrochim. Acta* **2021**, *381*, 138245.
4. Peng, Z.; Huang, J.; Wang, Y.; Yuan, K.; Tan, L.; Chen, Y. Construction of a hierarchical carbon coated Fe_3O_4 nanorod anode for 2.6 V aqueous asymmetric supercapacitors with ultrahigh energy density. *J. Mater. Chem. A* **2019**, *7*, 27313-27322.

5. Li, L.; Jia, C.; Shao, Z.; Wang, J.; Wang, F.; Wang, W.; Wang, H.; Zu, D.; Wu, H. Fe_3O_4/nitrogen-doped carbon electrodes from tailored thermal expansion toward flexible solid-state asymmetric supercapacitors. *Adv. Mater. Interfaces* **2019**, *6,* 1901250.
6. Su, S.; Lai, L.; Li, R.; Lin, Y.; Dai, H.; Zhu, X. Annealing-assisted dip-coating synthesis of ultrafine Fe_3O_4 nanoparticles/graphene on carbon cloth for flexible quasi-solid-state symmetric supercapacitors. *ACS Appl. Energy Mater.* **2020**, *3,* 9379-9389.
7. Zhang, M.; Wu, X.; Yang, D.; Qin, L.; Zhang, S.; Xu, T.; Zhao, T.; Yu, Z.-Z. Ultraflexible reedlike carbon nanofiber membranes decorated with Ni-Co-S nanosheets and Fe_2O_3-C core-shell nanoneedle arrays as electrodes of flexible quasi-solid-state asymmetric supercapacitors. *ACS Appl. Energy Mater.* **2021**, *4,* 1505-1516.
8. Luo, Y.; Tang, Y.; Bin, X.; Xia, C.; Que, W. 3D porous compact 1D/2D Fe_2O_3/MXene composite aerogel film electrodes for all-solid-state supercapacitors. *Small* **2022**, *18,* 2204917.
9. Kumar, A.; Das, D.; Sarkar, D.; Patil, S.; Shukla, A. Supercapacitors with prussian blue derived carbon encapsulated Fe/Fe_3C nanocomposites. *J. Electrochem. Soc.* **2020**, *167,* 060529.
10. Khalafallah, D.; Miao, J.; Zhi, M.; Hong, Z. Confining self-standing $CoSe_2$ nanostructures and Fe_3C wrapped N-doped carbon frameworks with enhanced energy storage performances. *Appl. Surf. Sci.* **2021**, *564,* 150449.
